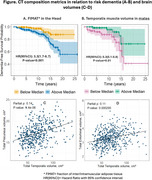# CT‐based Head & Neck Composition Metrics Predict Risk of Conversion to Early Dementia

**DOI:** 10.1002/alz70856_098937

**Published:** 2025-12-24

**Authors:** Farzaneh Rahmani, Joseph E. Ippolito, Jingxia Liu, Xu Yifei, Tammie L.S. Benzinger

**Affiliations:** ^1^ Mallinckrodt Institute of Radiology, St. Louis, MO, USA; ^2^ Mallinckrodt Institute of Radiology, Washington University in St. Louis, St. Louis, MO, USA; ^3^ Washington University in St. Louis, School of Medicine, St. Louis, MO, USA; ^4^ Washington University in Saint Louis, Saint Louis, MO, USA; ^5^ Washington University in St. Louis, St. Louis, MO, USA

## Abstract

**Background:**

Visceral adiposity, neck adiposity and sarcopenia are related to Alzheimer disease (AD) risk. Current amyloid confirmatory tests have the highest predictive value where there is high AD pretest probability. CT scan‐derived adipose and fat quantification can provide insights into this probability. We investigated the relationship between head CT composition metrics and risk of AD.

**Method:**

We selected participants with normal cognition (Clinical Dementia Rating Scale (CDRÒ=0) and negative amyloid PET at their baseline visit from the Knight Alzheimer Disease Research Center (Knight ADRC) cohort. Based on subsequent CDR measurements, we determined whether each participant: i) stably converted from normal cognition to early dementia (CDR>0) (*dementia converters*) or ii) remained cognitively normal throughout the follow up period (*healthy non‐converters*). The PET/CT scan closest to the last CDR=0 date was then used to extract the low‐dose attenuation correction CT scan necessary to perform CT composition analysis using the Data Analysis Facilitation Suite (DAFS, version 3) platform. Closest brain MRI within 1 year of this PET/CT scan was used for volumetric analyses through FreeSurfer 7.1.1. Survival and correlation analyses were used to test the relationship between head composition metrics and risk of conversion to early dementia and brain volumes, respectively.

**Result:**

Total of 500 participants met the inclusion criteria (age: 69±8, 41.8% male, 13.2% African American). Over a median follow up of 123 months, 48(2.6%) stably converted to early dementia. Individuals with higher fraction of inter‐intramuscular adipose tissue in the head have a 3‐times increased risk of conversion from normal cognition to early dementia (Figure A). Males, but not females, with higher normalized temporalis muscle volume were protected against conversion to early dementia (HR(95%CI):0.3(0.1‐0.8)), suggesting sex differences in this relationship (Figure B). Correlation analyses showed a direct relationship between the volume of the temporalis muscles and volume of the precuneus and entorhinal cortices that persisted even after correction for total intracranial volume (Figure C and D).

**Conclusion:**

Head and neck composition metrics inform future risk of dementia and correlate with brain atrophy suggesting a potential role for automated CT‐based screening paradigms based on this method.